# Hybridization capture sequencing for *Vibrio* spp. and associated virulence factors

**DOI:** 10.1128/mbio.00516-25

**Published:** 2025-06-25

**Authors:** Kyle D. Brumfield, Sana Enke, Brandon K. Swan, Lakeshia Carrasquilla, Michael D. Lee, David B. Stern, Linn Gieser, Nur A. Hasan, Moiz Usmani, Antarpreet S. Jutla, Anwar Huq, Katie Caviness, Jennifer S. Goodrich, Robert Bull, Rita R. Colwell

**Affiliations:** 1 University of Maryland Institute for Advanced Computer Studies, University of Maryland 1068 , College Park, Maryland, USA; 2 Maryland Pathogen Research Institute, University of Maryland 1068 , College Park, Maryland, USA; 3 National Bioforensic Analysis Center, National Biodefense Analysis and Countermeasures Center 189706 , Frederick, Maryland, USA; 4 Blue Marble Space Institute, Seattle, Washington, USA; 5 EzBiome Inc., Gaithersburg, Maryland, USA; 6 Department of Civil, Construction, and Environmental Engineering, University of Alabama 9968, Birmingham, Alabama, USA; 7 Geohealth and Hydrology Laboratory, Department of Environmental Engineering Sciences, University of Florida 3463, Gainesville, Florida, USA; 8 Federal Bureau of Investigation Laboratory Division, Scientific Response and Analysis Unit, Quantico, Virginia, USA; National Institutes of Health, Bethesda, Maryland, USA

**Keywords:** metagenomics, hybridization capture, targeted enrichment, whole-genome sequencing, next generation sequencing, microbiome, *Vibrio*, virulence

## Abstract

Proliferation of *Vibrio* spp. in aquatic ecosystems is associated with climate change and, concomitantly, increased incidence of vibriosis. They are autochthonous to aquatic environments globally, but traditional metagenomic methods for detecting and typing pathogenic *Vibrio* spp. are challenged by their presence in relatively low abundance and ability to persist in a viable but nonculturable state. In the study reported here, hybridization capture sequencing (HCS) was employed to profile low-abundance *Vibrio* spp. in environmental samples. The HCS panel targeted a family of molecular chaperones (CPN60) specific to 69 *Vibrio* spp. and 162 *Vibrio*-specific virulence factors. This approach was evaluated in parallel with traditional whole-community shotgun sequencing in a metagenomic analysis of water and oyster samples collected from the Chesapeake Bay. In addition, *Vibrio parahaemolyticus* and *Vibrio vulnificus* strains isolated from the samples were subjected to whole-genome sequencing to determine the genetic characteristics of pathogenic *Vibrio* spp. circulating in an aquatic environment. HCS, employed to determine the incidence and characterization of specific *Vibrio* spp., yielded significantly greater metagenomic insight, notably a variety of other *Vibrio* spp., including detection of *Vibrio cholerae*, *Vibrio fluvialis*, and *Vibrio aestuarianus*, in addition to *Vibrio parahaemolyticus* and *Vibrio vulnificus*, and also important virulence factors not detectable using traditional molecular methods. Thus, pathogenic *Vibrio* spp. in aquatic ecosystems may be far more common than currently understood. It is concluded that environmental surveillance should include HCS, a valuable tool for the detection and characterization of pathogenic agents in aquatic ecosystems, notably vibrios.

**IMPORTANCE:**

The increasing prevalence of pathogenic *Vibrio* spp. in aquatic ecosystems, driven by climate change, is closely linked to a rise in cholera and vibriosis cases, emphasizing the need for improved environmental surveillance. Vibrios are naturally occurring in aquatic environments globally, but traditional metagenomic methods for detecting and typing pathogenic *Vibrio* spp. are challenged by their presence in relatively low abundance and ability to persist in a viable but nonculturable state. In the study reported here, hybridization capture sequencing was employed to profile low-abundance *Vibrio* spp. in metagenomic samples, namely water and oysters collected from the Chesapeake Bay. This approach was evaluated in parallel with traditional whole-community shotgun sequencing and whole-genome sequencing of *Vibrio parahaemolyticus* and *Vibrio vulnificus* strains isolated from the samples. Results suggest pathogenic *Vibrio* spp. in aquatic ecosystems may be far more common than currently understood, when multiple methods are considered for environmental surveillance.

## INTRODUCTION

Members of the genus *Vibrio* are autochthonous to the aquatic environment and play an important role in carbon and nitrogen cycling as well as other significant biogeochemical processes (1, 2). While *Vibrio* spp. are present in larger numbers in coastal waters, their abundance is strongly influenced by environmental factors, notably temperature and salinity (3). Furthermore, *Vibrio* spp. are commensals and/or symbionts of aquatic invertebrates, namely crustaceans, zooplankton, and bivalves, all of which harbor these bacteria (3–6). *Vibrio* spp. have an established mutualistic relationship with zooplankton, namely copepods, a major host of these bacteria and considered a vector of *Vibrio cholerae* (5, 7, 8).

*Vibrio* spp. concentrate in oysters and other filter-feeding shellfish, which are often consumed raw, thereby exposing consumers to potentially infective doses of pathogenic agents (6, 9, 10). The Centers for Disease Control and Prevention (CDC) considers infections associated with any species of the family *Vibrionaceae* as reportable through the Cholera and Other *Vibrio* Illness Surveillance (COVIS) system (11). Historically, most infections in humans have been associated with only a few *Vibrio* spp., of which *V. cholerae*, *V. parahaemolyticus*, and *V. vulnificus* are significant (3, 11–13). Yet, there has been an increasing number of reports of vibriosis in humans and marine animals globally, with pathogenic strains of *Vibrio alginolyticus* and *Vibrio fluvialis* dramatically more relevant (3, 11, 13, 14). In the USA, the majority of reported *Vibrio* spp. infections have been foodborne (12). However, wound infections caused by *V. vulnificus* and related vibrios are rapidly outpacing foodborne infections in the USA (3, 11, 14, 15), corroborated by the increase in *Vibrio* spp. infections reported in Florida during 1992–2022 (15) and in Maryland during 2006–2019 (14). Concerningly, both global and regional climate variability are impacting the emergence and prevalence of these disease-causing agents, resulting in a trend of increased incidence of *Vibrio* spp. infections, which can be expected to expand further (8, 16, 17). Thus, routine monitoring and predictive intelligence models to assess and forecast the risk of vibriosis are critical to public health.

Traditionally, *Vibrio* spp. have been detected and characterized by using culture-dependent methods. A recent culture-based investigation of *V. parahaemolyticus* and *V. vulnificus* in the Chesapeake Bay, carried out between 2009 and 2022, reaffirmed specific environmental predictors for these bacteria and documented their population increase and extended seasonality, notably increased numbers during the fall months of the year (4). However, since the vast majority of microorganisms present in the environment have not been successfully cultured (18), relying solely on culture-based techniques would fail to detect a significant portion of microbial diversity, including *Vibrio* spp. that can enter a viable but nonculturable (VBNC) state (19). Molecular methods of detection, e.g., polymerase chain reaction (PCR) and loop-mediated isothermal amplification (LAMP), have been successfully employed for the identification of *Vibrio* spp. in both environmental and clinical settings (15, 20–25). However, due to the complex nature of the matrices that *Vibrio* spp. inhabit, e.g., water, sediment, and/or crustaceans and bivalves, even these molecular-based methods are not sufficient to assess the totality of their presence (26). The methods are also limited in genetic profiling of samples since they detect only one or a few markers in a single assay. Whole-genome sequencing (WGS) provides valuable insight into the phylogenetic diversity of *Vibrio* spp. (27, 28) but requires preparation of DNA from isolates in pure culture, hence being dependent on successful culture. Metagenomics obviates the need for culturing microorganisms of interest by utilizing the genetic material of a sample to identify composition, thus allowing detailed comparison and exploration of microbial communities (29).

Metagenomics currently includes a variety of approaches for genomic profiling of the microbiota (Fig. S1), directly by whole-community shotgun metagenomic sequencing and targeted using amplicon sequencing and hybridization capture sequencing (HCS) (29, 30). In addition to 16S rRNA gene sequencing, other PCR amplification protocols have been successfully employed for detecting *Vibrio* spp., including assays targeting specific markers such as the vibriobactin utilization (*viuB*) protein-coding gene for *V. cholerae* (31), and the heat shock protein 60 (HSP60), commonly referred to as chaperonins, CPN60, or GroEL, for broader *Vibrio* genus identification (32). Multiplex PCR approaches have allowed for the simultaneous detection of multiple targets within a single reaction (33, 34), and amplicon sequencing has been used in smaller prokaryotic genomes, namely viruses (35). However, these PCR-based methods are limited in scalability since they generally target only a small number of loci per assay and may miss genetically diverse or novel strains due to primer bias or low abundance in complex samples. Furthermore, PCR multiplexing is constrained by primer compatibility, making it impractical to screen for many diverse targets simultaneously.

Considering these various methods for detection and characterization, shotgun metagenomics is attractive in terms of determining relative abundance of members of the microbiome and their functional genes, including virulence and antimicrobial resistance determinants. Shotgun metagenomic sequencing has been successful in the detection of *Vibrio* spp. in clinical settings, namely determining pathogenic strains in the stool of cholera (36) and vibriosis patients (37). *Vibrio* spp. detection in wastewater employing shotgun metagenomics has also been successful (38, 39), and a few studies detected pathogenic vibrios in surface water (15, 40, 41). However, those studies showed limited genetic profile characterization of *Vibrio* spp. present in the samples. Hence, shotgun metagenomic sequencing for the identification of *Vibrio* spp., especially pathogenic strains, in environmental samples is limited since they are present in low abundance, thereby underestimating risk.

HCS amplifies specific genomic regions of interest, targeted prior to sequencing, to increase detection of low-abundance targets in complex samples. HCS employs microarray technologies for sequencing, whereby a fragmented shotgun library is selectively enriched by hybridization of nucleic acid fragments (DNA or RNA) representing multiple genomic targets. Generally, probes designed for HCS (ca. 80 bp–120 bp) are longer than for PCR (ca. 15 bp–20 bp), allowing for targets to be amplified even if mutations have occurred in the binding area. A major benefit of HCS over PCR amplicon sequencing is that it is not restricted by the overall size or number of targets; hence, there is less interference between capture probe sets, as with PCR primers.

Vezzulli et al. (42) employed HCS for direct genotyping and metagenomic analysis of low-abundance *V. cholerae* DNA in complex environmental samples based on biotinylated baits for enrichment of *V. cholerae* metagenomic DNA via hybridization (43). Additional hybridization capture bait sets have been designed for *Vibrio* spp. to study pathobiota of oysters (44) as well as 16S rRNA (45) and antimicrobial resistance genes (46). These studies concluded that without targeted sequencing, it would not have been possible to detect the broad set of *Vibrio* subvariants and associated biomarkers, as traditional methods lack the necessary scalability and resolution.

Building on previous work (42, 44–46), the objective of the current study was to provide accurate analysis of the composition of *Vibrio* communities in complex samples by development of HCS that allows determination of *Vibrio* spp. diversity, detection of pathogenic subspecies, and carriage of clinically relevant virulence factors (VFs). We describe HCS targeting 69 *Vibrio* spp. and 162 virulence markers. A comparison of this method with traditional shotgun metagenomics used for microbiome profiling of water and oyster samples collected from the Chesapeake Bay in 2019 is provided. With the addition of WGS analysis of *V. vulnificus* and *V. parahaemolyticus* isolates, it is concluded that by incorporating HCS as a complement to culture and related molecular techniques, enhanced characterization and improved understanding of *Vibrio* spp. in complex samples are achieved, providing valuable information for risk assessment.

## MATERIALS AND METHODS

### Site description and sample collection

Methods employed for sample collection and processing have been described in detail previously (4, 15), and a summary of methods relative to this study is provided here. Between June and October of 2019, water and oyster (Eastern oyster [*Crassostrea virginica*]) were collected at six stations in the Chesapeake Bay, Maryland, USA. Sampling event details and station abbreviations are shown in Table 1.

**TABLE 1:** Sampling location information

Station	Abbrev	Collection date (yr-mo-day)	Latitude	Longitude	Temp (°C)	Salinity (ppt)	pH	Conductivity (mS/cm)	Optical dissolved oxygen (mg/L)	Total Chl *a* (mg/L)	Pheophytin (mg/L)	Active Chl *a* (mg/L)
Chester River	CHE	2019-06-03	39.085980	−76.164233	25.2	3.7	7.32	6816	8.0	21.76	9.27	16.59
St. Mary’s River	SMR	2019-07-02	38.145361	−76.437833	26.6	9.1	8.6	15700	8.3	20.72	4.02	18.46
Manokin River (Tangier Sound)	MAN	2019-07-16	38.109861	−75.869111	29.7	12.5	8.03	21030	6.8	15.59	3.18	13.80
Broad Creek	BRO	2019-08-19	38.747250	−76.248167	29.2	8.8	8.2	ND^*a*^	6.5	16.49	6.79	12.70
Wicomico River	WIC	2019-09-10	38.277139	−76.826333	25.6	11.3	8.4	ND	ND	16.88	3.82	14.73
Miles River	MIL	2019-10-24	38.842556	−76.232417	16.8	14.2	8.1	23440	9.7	12.05	2.75	10.50

^*a*^ ND, not determined.

During each sampling event, environmental parameters, including water temperature, pH, dissolved oxygen (DO), salinity, conductivity, and total dissolved solids, were measured 0.3 m below the surface using a handheld water probe (Eureka, TX). Water (12 L) was collected 0.3 m below the surface using a Van Dorn water sampler (WildCo, NY) and stored in a sterile Nalgene carboy (Thermo Fisher Scientific, Waltham, MA). Up to 30 oysters were collected by dredging and stored in clean double-zipper freezer bags. Samples were transported to the laboratory in a cooler with ice, and the temperature of the coolers was monitored using a single-trip temperature alert indicator (LogTag Recorders, Auckland, New Zealand). The temperature of the water samples was recorded upon arrival at the laboratory, and samples were stored at 4°C until processing (<4 h).

### Sample processing

Water (250 mL) was filtered using 25 mm glass microfiber filters (Cytiva, MA), and concentrations of chlorophyll-*a* (total and active) and pheophytin were measured in acetone extracts on a Shimadzu UV 2401PC spectrophotometer, following procedures recommended by University of Maryland Center for Environmental Science for fluorometric detection of chlorophyll-*a* in waters of fresh/estuarine/coastal areas (47). Chlorophyll-*a* analysis was performed in duplicate, and averages are presented.

Water and oyster samples were treated following methods outlined in the Bacteriological Analytical Manual for food sampling/preparation of sample homogenate (48) and *Vibrio* (49). From each sampling event, 250 mL of water was concentrated using syringe filtration with 0.22 µm pore size Sterivex filter units (Millipore Sigma, MA). Oysters were rinsed and scrubbed under running deionized water to remove debris from the shell and opened using a sterile shucking knife. Oyster tissue in an equal volume of phosphate-buffered saline (pH 7.4) was homogenized in a sterile blender for 90 s. Filter units and homogenized oyster tissue (500 µL) were stored at −80°C in DNA/RNA Shield Stabilization Solution (Zymo Research, CA).

### Isolation of *V. parahaemolyticus* and *V. vulnificus*

Water and oyster samples were respectively inoculated using alkaline peptone water (APW; 1% peptone, 1% NaCl [pH 8.5]). Briefly, unfiltered water (1 L) and homogenized oyster tissue (10 g) were resuspended in APW (10×) and incubated for 16 h at 37°C with moderate aeration (orbit diameter 2.5 cm × 30 rpm). A loopful of pellicle from each APW-enriched sample was respectively subcultured on selective agar media, including *Vibrio*-specific chromogenic agar (CHROMagar, France), thiosulfate citrate bile-salts sucrose agar (Oxoid, Canada), and M190 *V. vulnificus* agar (50), and incubated up to 18 h at 37°C. Presumptive *Vibrio* spp. colonies were purified on Luria-Bertani agar (Difco, NY) and maintained under standard bacteriological conditions for *Vibrio* spp. (51). Confirmation and identification of *Vibrio* spp. were done using established molecular assays, as detailed below.

### Nucleic acid preparation

Genomic DNA was prepared from homogenized oyster tissue and pure culture isolates grown under standard conditions (Luria-Bertani broth with aeration at 37°C for 16 h), using the DNeasy Blood and Tissue Kit (Qiagen, Germany). Total DNA was isolated from the microbial biomass collected on Sterivex filter units, using the ZymoBIOMICS DNA Miniprep Kit (Zymo Research, CA) with modifications recommended previously (52). DNA extracts were further purified using the DNA Clean and Concentrator Kit (Zymo Research, CA).

### Preparation of mock community

To prepare the mock community, DNA was prepared from pure cultures of 15 representative *Vibrio* spp. type strains (*V. cholerae* O39 AM-19226, *V. cholerae* O1 classical O395, *V. cholerae* O1 El Tor N16961, *V. cholerae* O139 MO10, *V. vulnificus* [*vcgE*] ATCC 27562, *V. vulnificus* [*vcgC*] ATCC 29307, *V. parahaemolyticus* O3:K6 VP-NY4, *V. parahaemolyticus* ATCC 17802, *V. alginolyticus* ATCC 17749, *Vibrio mimicus* ATCC 33653, *Vibrio furnissii* ATCC 35016, *V. fluvialis* ATCC 33809, *Vibrio harveyi* ATCC 14126, *Vibrio aestuarianus* ATCC 35048, and *Aliivibrio* [*Vibrio*] *fischeri* ATCC 25918), using the DNeasy Blood and Tissue Kit (Qiagen, Germany). Double-stranded DNA concentrations were measured using the NanoDrop ND-1000 Spectrophotometer (Thermo Fisher, MA), and genomic DNA from each strain was normalized with nuclease-free water to equal concentration (1,000 ng) and combined. Pooled mock community DNA was purified using the DNA Clean and Concentrator Kit (Zymo Research, CA).

### Polymerase chain reaction

PCR methods have previously been established for detection and characterization of *Vibrio* spp. Primer sequences, amplicon sizes, and thermal cycler conditions are detailed in Table 2. Amplified products were fractionated by electrophoresis through 1.5% (wt/vol) agarose gel along with a 100 bp molecular weight marker (HyperLadder; BioLine, NJ) and visualized using SafeGLO Pre-Stain (BioLink, CA). For quality control, a no template control (NTC) consisting of nuclease-free water and positive/negative controls was included with each reaction.

**TABLE 2:** Primers, PCR parameters, and reference strains used in this study

Description (reference)	Oligonucleotide name	Sequence (5´−3´)	PCR product size (bp)	Annealing temp (°C)	Reference
*Vibrio* genus 16S rRNA	567F	GGCGTAAAGCGCATGCAGGT	120	55	(21)
	680R	GAAATTCTACCCCCCTCTACAG			
*toxR* of *V*. *parahaemolyticus* (Vp), *V*. *cholerae* (Vc), and *V*. *vulnificus* (Vv)	UtoxF	GASTTTGTTTGGCGYGARCAAGGTT			(53)
	Vp-toxR	GGTTCAACGATTGCGTCAGAAG	297 (*Vp*)	60	
	Vc-toxR	GGTTAGCAACGATGCGTAAG	640 (*Vc*)	55	
	Vv-toxR	AACGGAACTTAGACTCCGAC	435 (*Vv*)	55	
Total and hemolysin-producing *V*. *parahaemolyticus* via *tlh*, *tdh*, and *trh*	L-tl	AAAGCGGATTATGCAGAAGCACTG	450 (*tlh*)	58	(20)
	R-tl	GCTACTTTCTAGCATTTTCTCTGC			
	L-tdh	GTAAAGGTCTCTGACTTTTGGAC	269 (*tdh*)	58	
	R-tdh	TGGAATAGAACCTTCATCTTCACC			
	L-trh	TTGGCTTCGATATTTTCAGTA	500 (*trh*)	58	
	R-trh	CATAACAAACATATGCCCATTTCCG			
*V*. *vulnificus* hemolysin *vvhA*	F-vvh	TTCCAACTTCAAACCGAACTATGAC	205	58	(54)
	R-vvh	ATTCCAGTCGATGCGAATACGTTG			
*V. vulnificus* biotype 1 virulence-correlated gene *vcgC/E*	F-vcgC	AGCTGCCGATAGCGATCT	97 (*vcgC*)	57	(55)
	R-vcgC	TGAGCTAACGCGAGTAGTGAG			
	F-vcgE	CTCAGAAAGGCTCAATTGAC	199 (*vcgE*)	57	
	F-vcgE	GATTAACGCTGTAAGGCCG			
*V. vulnificus pilA*	VvpAF32	TGGCTGCTGTTGCTATTC	217	60	(56)
	VvpAR2	GGTCCACCACTAGTACCAAC			
*V. vulnificus rtxA1*	ERMGST1	CGGGATCCTATGGCGTGAACGGCGAAG	1,440	68	(57)
	ERMGST2	CGGGATCCAGCAGCCACAAGCGATTC			
Toxigenic *V*. *cholerae* via *ctxA*, *rfb-*O1, and *rfb*-O139	O139F2	AGCCTCTTTATTACGGGTGG	449 (O139)	55	(58)
	O139R2	GTCAAACCCGATCGTAAAGG			
	O1F2	GTTTCACTGAACAGATGGG	192 (O1)	55	
	O1R2-2	GGTCATCTGTAAGTACAAC			
	VCT1	ACAGAGTGAGTACTTTGACC	308 (*ctxA*)	55	
	VCT2	ATACCATCCATATATTTGGGAG			

### Next-generation sequencing (NGS)

Samples analyzed by WGS include seven isolates (*V. parahaemolyticus*, *n* = 3; *V. vulnificus n* = 4) from environmental water samples collected in 2019 (4) and identified by PCR as pathogenic *V. parahaemolyticus* ([Vp-*toxR*^+^ and *tlh*^+^] and [*tdh*^+^ and/or *trh*^+^]) or *V. vulnificus* ([Vv-toxR^+^, *vvhA*^+^, and *vcgE*^+^] and [*pilA*^+^ and/or *rtxA*^+^]). Microbiome analysis included Sterivex concentrated water and homogenized oyster tissue from each of the six sampling events during 2019 (12 total samples, consisting of six water and six oyster samples), along with a mock community sample (positive control) and NTC consisting of nuclease-free water processed using the DNeasy Blood and Tissue Kit (Qiagen, Germany). WGS and shotgun metagenomic libraries were prepared using the NEBNext Ultra II FS Library Prep Kit for Illumina sequencing (New England Biolabs, MA). For HCS, target enrichment was performed in four-plex on an aliquot of each of the libraries prepared for shotgun metagenomic sequencing, using the xGen Hybridization Capture of DNA Libraries kit (IDT, IA) with custom probes, described below.

Double-stranded DNA concentration was measured using the Qubit 3.0 fluorometer (Thermo Fisher, MA) and confirmation of library size was achieved using High Sensitivity D1000 ScreenTape Assay (Agilent Technologies, CA). NGS was done using the NextSeq 2000 System (Illumina, CA) with 150 bp paired-end reads. WGS and shotgun metagenomics were performed targeting 40M paired read clusters, while HCS was done targeting 10M. To avoid laboratory contamination of test samples, all analyses, including DNA extraction, amplification, and library preparation, were carried out in a separate Good Laboratory Practice-accredited and Current Good Manufacturing Practice-compliant laboratory (EzBiome Inc., MD) using a dedicated set of reagents and consumables. While DNA extractions for NTCs and mock community positive controls were performed separately from test samples, DNA amplification and library preparation were conducted simultaneously to ensure proper sequencing controls.

### Probe design

Molecular chaperones are present in plastids, mitochondria, and cytoplasm of eukaryotes, bacteria, and archaea. Group I chaperonins (CPN60, also known as HSP60 or GroEL) are a diverse family of molecular chaperones specific to bacteria and have proved useful for differentiating closely related taxa and are commonly employed as targets for detection and identification of species, including vibrios (32, 59–61). An investigation of CPN60 sequences from bacterial and eukaryotic species (62) led to the discovery of a conserved region (~550 bp–570 bp) termed “universal target” (UT) to delineate closely related taxa and provide greater phylogenetic resolution than 16S rRNA, by providing a higher evolutionary rate of CPN60 (63–66). To this end, a reference database was created by downloading all nonredundant nucleotide sequences for the CPN60 UT (one sequence per species, type strain preferred) from the manually curated chaperonin database (67). Members of the genus *Vibrio* were extracted (*n* = 70) and aligned using the CLUSTALW algorithm (68), and evolutionary history was inferred using the maximum-likelihood method and Tamura-Nei Model (69) in MEGA 11 v.11.0.10 (70). To ensure phylogenetic resolution in the reference database, indistinguishable reference sequences were omitted. Two nodes (*Vibrio agarivorans* and *Vibrio sagamiensis*) were indistinguishable, hence combined, i.e., *V. agarivorans*/*V. sagamiensis*, resulting in 69 unique CPN60 UT sequences. In addition to the CPN60 sequences, relevant genomic markers (a total of 162 genes) commonly used for *Vibrio* spp. detection and identification, along with specific virulence factors, were manually curated from public repositories, namely Virulence Factor Database (79 genes) (71) and NCBI Pathogen Detection Reference Gene Catalog (83 genes) (72). CPN60 UT sequences and additional genomic targets were evaluated using CD-HIT-EST v.4.8.1 (73) to eliminate identical sequences in the reference database. The resulting targets comprised 231 unique reference sequences (~281 kbp) (Table S1).

Custom probes (120 bp DNA oligonucleotide sequences) were designed commercially by Integrated DNA Technologies xGen Pool Design Service (IDT, IA). The initial design contained 2,450 unique probes (2,553 probes pre-duplicate removal) (Table S2). Coverage of the original probe design was evaluated by mapping probes back to the reference database using Bowtie2 v.2.4.1 (74) with the “--very-sensitive” option. To make the HCS panel more accessible and cost-effective, the overall number of probes was reduced based on similarity of original target sequences by removing predicted functionally redundant probes, using a multi-strain algorithm (IDT, IA). The output of the multi-strain design contained 1,779 unique probes, considered functionally equivalent to a 1× tiling design (Table S3). Probe sequences generated using the multi-strain algorithm were analyzed for potential homology with other taxa using Kraken2 v.2.1.3 (75) and Bracken (76) with the PlusPF database, which includes reference sequences of archaeal, bacterial, viral, plasmid, protozoan, fungal, human, and vector origin, and visualized using Krona Tools (77).

Resulting probe sequences of multi-strain design were synthesized with 5´ biotin modification using xGen Minimal Residual Disease Research Hybridization Panel Tool (IDT, IA). The xGen Universal Blockers (IDT, IA) were employed to prevent off-target fragments from annealing to intended target sequence via adapter-to-adapter hybridization. HCS libraries were prepared using 500 ng from each of the 12 sequencing libraries prepared for shotgun metagenomics.

### Preprocessing of sequencing reads

Read quality was assessed using FastQC v.0.12.1 (78) and MultiQC v.1.11 (79). Adapter sequences were removed, and low-quality bases were trimmed using fastp v.0.23.4, with options for base correction, polyG tail trimming, and low-complexity filtering enabled (80). While unintended human DNA contamination in sequencing read libraries was not expected, it has been shown to be a major problem in genomics, resulting in false identification of spurious proteins (81). Accordingly, potential reads originating from human DNA were identified and removed from all libraries using Bowtie2 (74) by mapping reads against the Human Telomere-to-Telomere Consortium CHM13 Project reference genome (GCA_009914755.4). For microbiome samples, Bowtie2 was used to remove potential reads originating from oyster DNA by mapping reads against reference genomes of the Eastern oyster (*Crassostrea virginica*: GCA_002022765.4) and Pacific oyster (*Magallana gigas*: GCA_963853765.1). To minimize the impact of background contamination on microbiome data integrity, notably in low microbial biomass samples (82), we employed a conservative approach to identify and remove potential contaminating reads (Fig. S2). Sequencing reads from NTCs were first assembled into contigs using the metagenomics workflow from Bioinformatics Tools (bit) v.1.9.4 (83). Sample read libraries were then mapped to these NTC-derived contigs using Bowtie2 (74), and any matching reads were excluded from downstream analyses.

### Comparative genomics of *Vibrio* spp. isolates

Processed sequencing read libraries of purified culture isolates were assembled into contigs using Unicycler v.0.5.0, a SPAdes-based optimizer for bacterial genome assemblies (84). Contigs less than 500 bp were discarded. Assembly statistics, completeness, and genome quality were assessed using the Quality Assessment tool for Genome Assemblies v.5.2.0 (85) and Benchmarking Universal Single-Copy Orthologs (BUSCO) tool v.5.7.1 (86) with 1,445 BUSCOs from the “vibrionales_odb10” data set. Draft genome assemblies were annotated with Prokka v.1.14.6 (87), using all complete reference genomes of the genus *Vibrio* (*n* = 67) and all complete genomes of *V. cholerae* (*n* = 109), *V. vulnificus* (*n* = 27), and *V. parahaemolyticus* (*n* = 113), from RefSeq as guide, accessed 4 June 2024. Presence of antimicrobial resistance genes and virulence factors was assessed using the Short, Better Representative Extract Dataset (ShortBRED) algorithm (88) and BLAST (89), as described below. Multilocus sequence types (MLSTs) were predicted using the MLST software program (90) with the Public Database for Molecular Typing and Microbial Gene Diversity (PubMLST) database (91). Isolates identified as *V. parahaemolyticus* were subjected to serotyping by profiling serogroup-specific genes based on WGS data, using VPsero (92).

### Phylogenomic tree reconstruction

Genomic data in public repositories vary widely in quality, including differences in genome size, number of contigs, assembly status (i.e., complete, chromosome, scaffold, contig), and N50 values. These inconsistencies may introduce bias during whole-genome alignment, as higher quality and more complete genomes may disproportionately influence phylogenetic analyses. To mitigate this issue, genome-level evolutionary relationships among the *Vibrio* spp. were inferred using GToTree v.1.8.6 (93), which constructs phylogenies based on a core genome approach using single copy genes (SCGs). First, a local database was built from CDSs of (*n* = 233) representative *Vibrio* spp. genomes present in the Genome Taxonomy Database (GTDB) v.220 (94). Using the “gtt-gen-SCG-HMMs” command within GToTree, all protein family (Pfam) Hidden Markov Model (HMM) profiles were downloaded from the Pfam database, released 28 May 2024 (95), and HMMs with an average coverage of <50% of the underlying protein sequence were removed. The filtered Pfam HMM profiles were searched against all coding sequences from the representative *Vibrio* spp. database using HMMER v.3.4 (96), specifically “hmmsearch” with the “-cut_ga” flag. Pfams with exactly one hit in at least 90% of the representative *Vibrio* spp. genomes were retained as the SCG set for the genus *Vibrio* (*n* = 355 targets). For building phylogenetic trees, CDSs were retrieved for input genomes from public repositories or called with Prodigal v.2.6.3 (97) when CDSs were not available. The *Vibrio*-specific SCGs were identified using “hmmsearch” as mentioned, and spurious gene hits were filtered based on the fraction of target genes detected. Each gene set was aligned with MUSCLE v.5.1 (98), and automated trimming was done with TrimAl v.1.4.15 (99). The trimmed gene sets were concatenated for each genome, and phylogenetic trees were built using FastTree v.2.1.11 (100), employing the maximum-likelihood Jones-Taylor-Thornton model with the default number of rate categories (CAT). The resulting trees were viewed with iTOL v.6 (101). Using this approach, the genomic relatedness of seven *Vibrio* spp. isolates from this study was evaluated among the 233 representative *Vibrio* spp. from GTDB (Table S4). These isolates were further classified with existing lineages of *V. parahaemolyticus* (*n* = 259) (Table S5) and *V. vulnificus* (*n* = 118) (Table S6).

### Metagenomic community profiling

A total of 12 samples (both water and oyster from six sampling events) and controls (mock community positive control and NTC) were selected for metagenomic community profiling, which included both shotgun metagenomic sequencing and HCS.

### Whole-community microbiome profiling

Metagenomic sequencing reads were profiled using KMCP v.0.9.4 (102) with multiple prebuilt databases from the KMCP software suite, including GTDB v.214 (94) (bacteria and archaea), RefSeq (fungi), and GenBank (viruses), using “single-end” mode for higher sensitivity, per recommendations of the KMCP documentation. The search results were merged, and profiling was done with flags for “--mode 3” and “--no-amb-corr.” Resulting KMCP scores, i.e., 90th percentile of query coverage (matched kmers/query kmers), were filtered, and taxa with a score <100 were labeled “unclassified.” Taxonomic profiles were visualized using Phylosmith v.1.0.6 (103) and presented as relative sequencing read abundance.

Depth of the sequencing read libraries was normalized to the sample with the minimum number of sequencing reads >10,000 using the “rarefy_even_depth” function of phyloseq v.1.46 (104); four oyster samples (two each from HCS and shotgun metagenomics) were omitted because they contained fewer reads than the simulation threshold. Alpha diversity metrics were obtained using phyloseq’s “estimate_richness” function. The observed (number of taxa), Chao1 (richness), and inverse Simpson’s diversity index (richness under uniform evenness) were visualized using the “plot_richness” function of phyloseq (104).

### Gene profiling for CPN60 diversity

Processed sequencing read libraries of metagenomic samples were assembled into contigs using the workflow for metagenomics from Bioinformatics Tools (bit) v.1.9.4 (83). To assign taxonomy to the CPN60 sequences, a custom BLAST (89) database was built using the full nucleotide sequences of all group I reference taxa present in the chaperonin database, accessed 19 August 2024 (67). Metagenomic contigs were mapped against the CPN60 database using blastn v.2.16.0 (89) with an e-value threshold of 1E − 180. Taxonomy was assigned initially at the species level for positive hits with ≥90% identity and ≥30% query coverage. To confirm taxonomic identity, the evolutionary relationships of identified CPN60 sequences were explored among *Vibrio* spp. reference gene sequences present in the chaperonin database (67). Briefly, nucleotide sequences coding for CPN60 were extracted from metagenomic contigs (*n* = 10) (Table S7) and aligned with reference gene sequences (*n* = 21) (Table S8) using Clustal Omega v.1.2.4 (105), and evolutionary history was inferred using the neighbor-joining method (106) in MEGA 11 v.11.0.10 (70) with 1,000 bootstrap replications. Resulting trees were visualized using iTOL v.6 (101).

### Detection of antimicrobial resistance and virulence determinants

Presence of antimicrobial resistance genes and virulence determinants was determined for both *Vibrio* spp. isolates and metagenomic samples using ShortBRED v.0.9.5 (88) and blastx v.2.16.0 (89). Briefly, the ShortBRED pipeline consists of two components: (i) a method that reduces reference proteins of interest to short, highly representative amino acid sequences, i.e., “markers” and (ii) a search step that maps reads to these markers to quantify relative abundance of their associated proteins (88). Pre-computed ShortBRED markers were employed for resistance-related determinants targeting the Comprehensive Antibiotic Resistance Database (CARD) 2017 (107), and custom markers were designed to target virulence factors. Genes from the Virulence Factor Database (VFDB) 2025 (71), supplemented with those used in probe design of the HCS panel (Table S1), served as the proteins of interest for identifying marker families using “shortbred_identify.py” with the “--clustid 0.95” option. The Comprehensive and Non-redundant UniProt Reference Clusters (Uniref90) database (108), accessed 27 February 2024, was used as the reference proteins to help rule out non-unique regions of the proteins of interest. ShortBRED will record a hit to a marker if the resulting alignment has at least 95% identity and is at least as long as the minimum of the marker length or 95% of the read length. Gene abundances were normalized in reads per kilobase per million mapped reads (RPKM) using “shortbred_quantify.py.” That is, genes detected in a sample were normalized to the total number of reads in a sample and the gene length. In addition, assembled contigs were mapped against BLAST databases, built using protein sequences of the antimicrobial resistance and virulence determinants, using blastx (89) with an e-value threshold of 1E − 180. Genes were considered to be present only if detected by both methods.

## RESULTS

### Environmental parameters and PCR of *Vibrio* spp.

**Fig 1:**
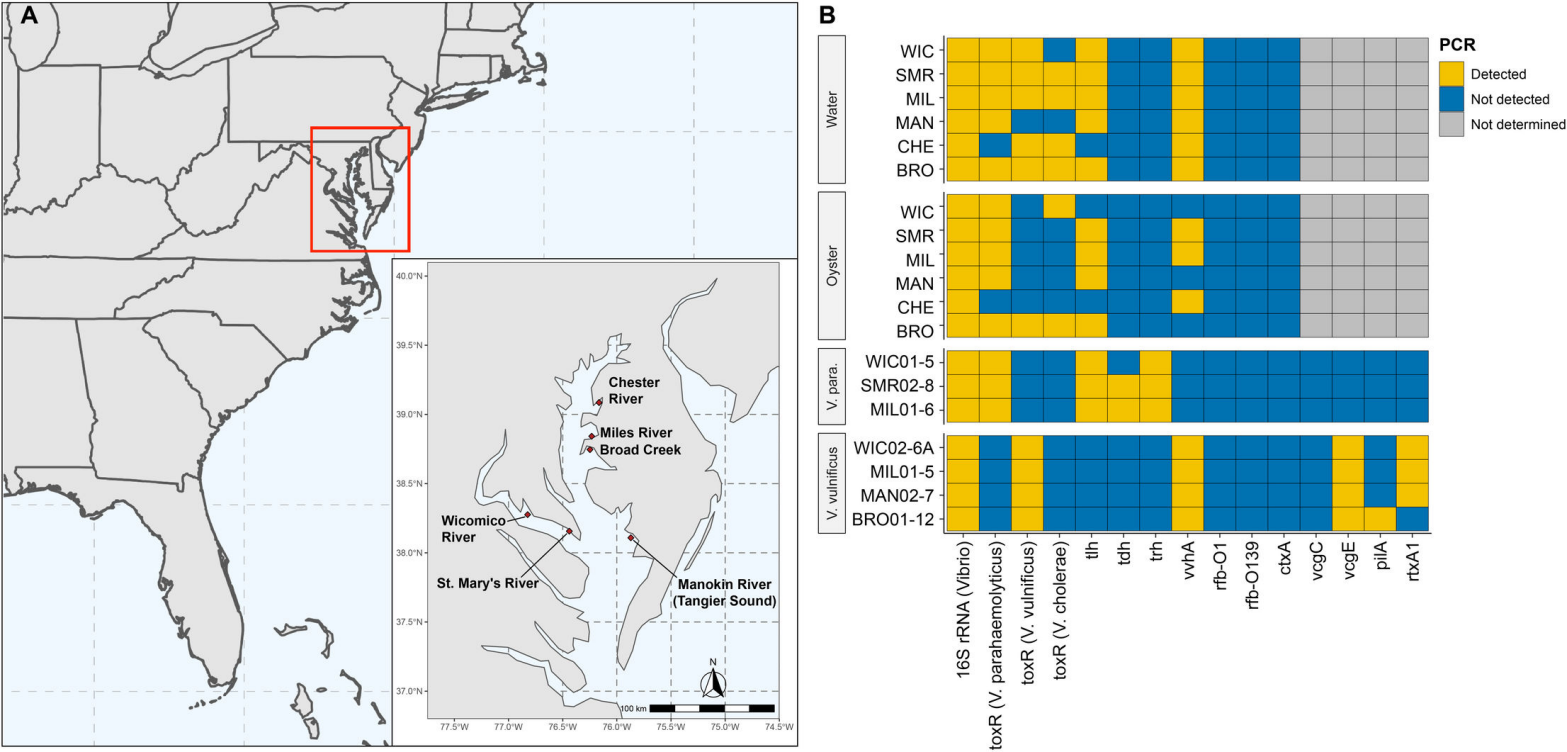
Area of study and PCR characterization. (**A**) Map of sampling locations in the Chesapeake Bay, Maryland, where water and oyster samples were collected between June and October 2019. (**B**) Heatmap showing detection (presence/absence) of *Vibrio* genus- and species-specific biomarkers, including virulence-associated genes, in environmental water and oyster samples as well as pure culture isolates (*V. parahaemolyticus* and *V. vulnificus*).

Between June and October of 2019, samples were collected from six stations (Fig. 1A). A summary of environmental parameters recorded during each sampling event is presented in Table 1. Temperature did not vary significantly across stations at the time of collection but increased with seasonality as expected, with the warmest temperatures observed during July and August. In general, lower salinity concentrations were recorded at stations located in the upper bay, namely CHE, MIL, and BRO, compared to those in the lower bay.

Detection of genetic markers targeting the genus *Vibrio*, *V. parahaemolyticus*, *V. vulnificus*, and *V. cholerae*, as well as important virulence factors, was achieved using PCR. Reaction parameters are shown in Table 2 and results in Fig. 1B. Presence of the genus *Vibrio* (16S rRNA) was detected in all samples. *V. vulnificus* (*toxR* and/or *vvhA*) was detected at all stations, *V. parahaemolyticus* (*toxR* and/or *tlh*) was detected at all stations except those from CHE, and *V. cholerae* (*toxR*) at all stations except MAN. Pathogenic biomarkers for *V. parahaemolyticus* (*tdh* and *trh*) and *V. cholerae* (*rfb*-O1, *rfb*-O139, and *ctxA*) were not detected in the environmental samples. However, *V. parahaemolyticus* isolates recovered from these stations were shown to carry *tdh/trh*. Of the three *V. parahaemolyticus* isolates, all carried *trh,* and two carried both *trh* and *tdh*. Of the four *V. vulnificus* isolates, all encoded the environmental *vcgE* biotype, three encoded *rtxA*, and one encoded *pilA*.

### Microbiome analysis

#### Validation on a microbial mock community

Rather than sequencing all genetic material in a sample, as done in traditional shotgun metagenomic sequencing, targeted HCS selectively amplifies specific genomic targets of interest prior to sequencing. To this aim, a targeted HCS protocol was developed for analysis of *Vibrio* spp. diversity and detection of primary virulence factors. A total of 1,779 probes were designed (Table S3) to target 69 *Vibrio* spp. and 162 virulence determinants (Table S1). To assess specificity, probe sequences were profiled using Kraken2 (75) and Bracken (76) against the PlusPF database, curated to include archaea, bacteria, viruses, plasmids, protozoa, fungi, human, and vectors. The results showed minimal homology with non-target taxa, with >99% of probe sequences identified as originating from *Vibrio* (Fig. S3), indicating that probes were appropriately designed for the intended application.

Hybridization capture sequencing and shotgun metagenomics were then used to profile a synthetic metagenome composed of equal DNA concentrations from 15 representative *Vibrio* spp. type strains. These included four strains of *V. cholerae*, two strains each of *V. vulnificus* and *V. parahaemolyticus*, and one strain each of *V. alginolyticus*, *Vibrio mimicus*, *Vibrio furnissii*, *V. fluvialis*, *Vibrio harveyi*, *Vibrio aestuarianus*, and *Aliivibrio* (*Vibrio*) *fischeri*.

To calculate the proportion of reads corresponding to target genes, Bowtie2 (74) was used to map processed sequencing reads against the genes included in the HCS panel design (Table S1). The results demonstrated that HCS significantly enriched target genes, with approximately 96% of reads mapping to panel targets (94% corresponding to virulence determinants and 1.6% to *Vibrio*-specific *cpn* gene sequences). In contrast, shotgun metagenomics yielded less than 2% of the reads carrying target genes (1% virulence determinates and 0.8% *cpn* sequences). These findings indicate that HCS effectively enhances the detection of target genes compared to traditional shotgun metagenomics.

K-mer-based metagenomic profiling was performed on control samples using the KMCP algorithm with whole-genome reference databases (Fig. S4). Among the 10 representative *Vibrio* spp., 9 were correctly identified by shotgun metagenomics and 8 by HCS. In the latter case, *V. alginolyticus* was identified as *Vibrio diabolicus*, which is a taxonomic synonym for *V. alginolyticus. V. fischeri* was not detected using either method. Since the mock community was prepared with equal DNA concentrations for each strain, *V. cholerae* was expected to be the most abundant, followed by *V. parahaemolyticus* and *V. vulnificus* with the remaining species appearing at lower abundances. Shotgun sequencing most closely matched this expected profile, while HCS showed more than 60% relative sequencing read abundance profiled as *V. cholerae*. Importantly, no taxa were detected in the NTC samples for either HCS or shotgun metagenomics. Furthermore, all taxa identified in the mock community were classified as members of the genus *Vibrio*, indicating no detectable cross-contamination among samples.

#### Community microbiome profiles employing KMCP

**Fig 2:**
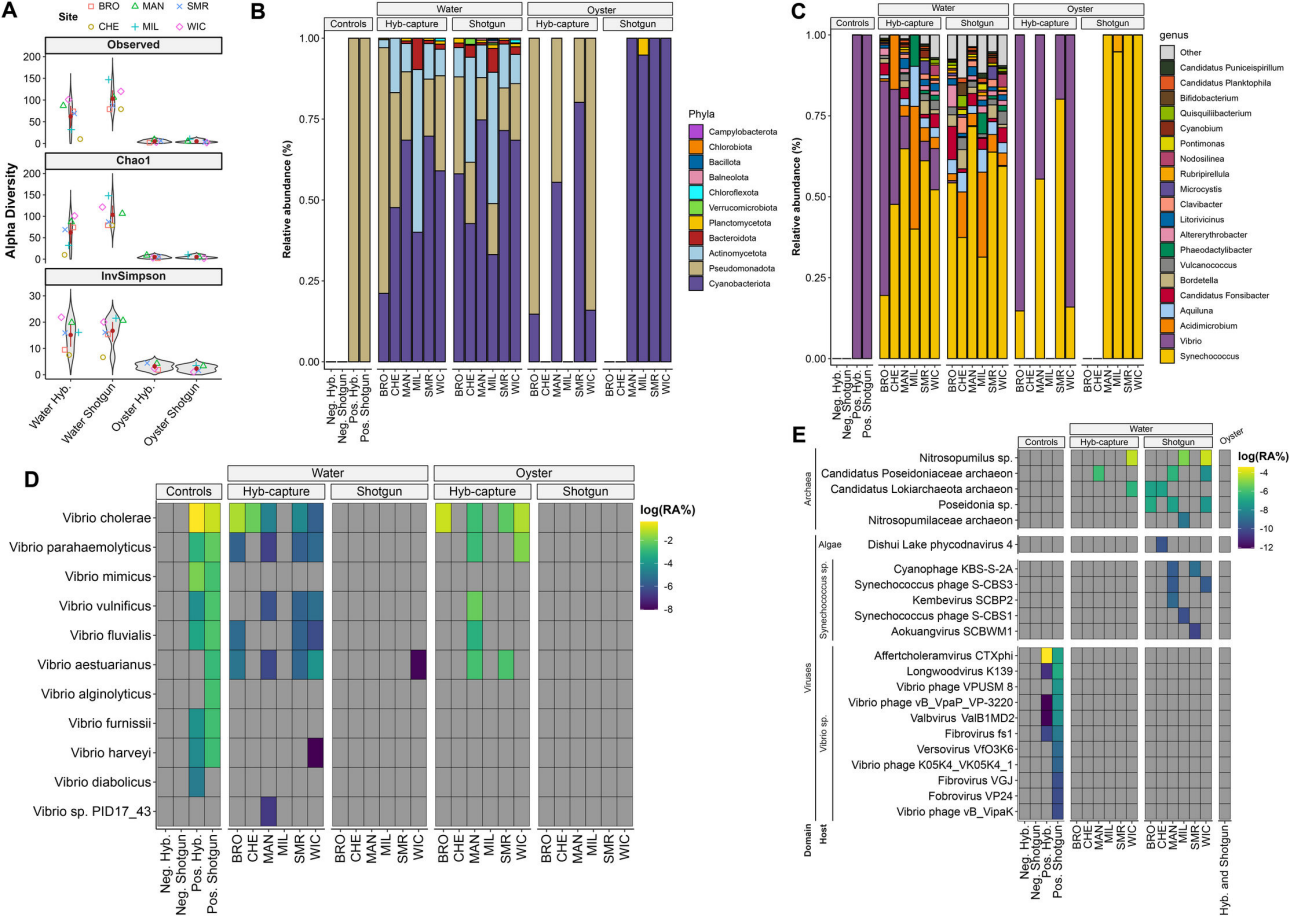
Microbial community composition based on k-mer alignment to whole-genome reference databases. (**A**) Alpha diversity metrics (observed, Chao1, and inverse Simpson indices) at the species level, comparing water and oyster microbiome profiled by hybridization capture sequencing and shotgun metagenomics. Violin plots are grouped by sample type and colored by sample site. (**B**) Stacked bar plots showing the relative sequence read abundance (%) of dominant bacterial and archaeal phyla across control, water, and oyster samples. (**C**) Stacked bar plots showing relative abundance (%) of the top 20 most abundant genera. (**D**) Heatmap of log-transformed relative abundances, log(RA%), of *Vibrio* spp. detected across samples and controls, highlighting improved detection sensitivity of hybridization capture sequencing compared to shotgun metagenomics. (**E**) Heatmap of log-transformed relative abundances, log(RA%), of archaeal, algal, viral, and phage taxa, showing broader taxonomic diversity captured by shotgun metagenomics in water samples. No archaeal, algal, viral, or phage taxa were found in oyster samples; these taxa were combined for each sequencing method (hybridization capture and shotgun metagenomics) to aid visualization. Taxonomic profiling was performed using KMCP with reference databases from GTDB, RefSeq, and GenBank.

Twelve samples, including water and oyster collected during six sampling events, were selected for microbiome analysis based on results from PCR and ability to recover *Vibrio* spp. isolates from the area. Subsequently, all samples were subjected to whole-community shotgun metagenomics and targeted HCS (Fig. 2). Measures of alpha diversity are shown in Fig. 2A. Overall, a greater number of microbial species were detected using shotgun metagenomic sequencing compared to HCS (Fig. S5). However, no significant differences were observed for alpha diversity indices between sequencing methods. Although it is worth noting that differences were observed between sample types, with overall alpha diversity significantly higher in water samples, compared to oyster, for both sequencing methods.

Community microbiome profiles were evaluated using the KMCP algorithm with whole-genome reference databases. Bacterial phyla detected were similar via both sequencing methods, i.e., HCS and shotgun metagenomics (Fig. 2B). *Cyanobacteriota* were dominant in oyster samples and also profiled in water samples. *Pseudomonadota* (formerly *Proteobacteria*) were most abundant in water samples and frequent in oyster samples profiled by HCS but not detected by shotgun metagenomics. Bacteria of the phyla *Actinobacteria*, *Bacteroidota*, and *Verrucomicrobiota* were detected in all water samples, regardless of sequencing method. A greater number of genera were profiled in water samples (Fig. 2C), compared to oyster. However, it is worth noting that for oyster samples, the majority of sequencing reads were removed during preprocessing as being profiled as *Crassostrea virginica* (Fig. S2). Of the reads profiled in oyster samples, *Synechococcus* was dominant, while *Acidimicrobium* was common in water samples. Members of the genus *Vibrio* profiled to species are shown in Fig. 2D. Employing HCS, *Vibrio* spp. were detected in five of six stations, including five water and four oyster samples. In comparison, *Vibrio* was detected in only one water sample and not in oyster samples, employing shotgun metagenomic sequencing. For HCS samples, *V. cholerae* was the most commonly detected species, followed by *V. parahaemolyticus*, *V. vulnificus*, *V. fluvialis*, and *V. aestuarianus*. While HCS showed increased sensitivity in detecting target taxa, namely members of the genus *Vibrio*, shotgun metagenomics proved best in detecting archaea and viruses not included in the HCS panel (Fig. 2E). Archaea and viruses were not detected in any of the oyster samples. Of those profiled in water samples, pathogenic viruses known to infect humans were not detected, with bacteriophages the majority, namely those specific to *Synechococcus* bacteria.

#### Taxonomic diversity of CPN60

**Fig 3:**
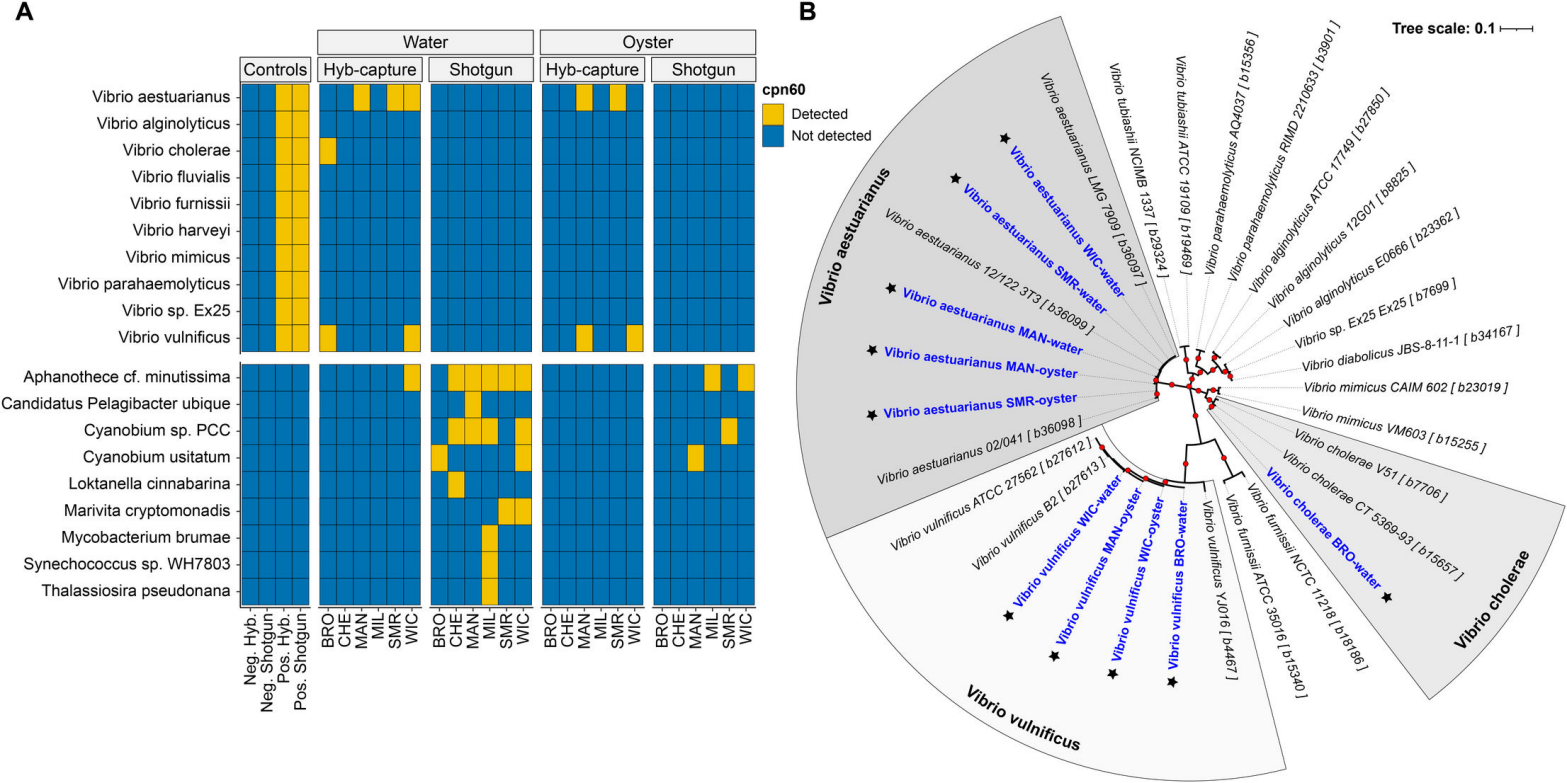
CPN60 diversity and phylogenetic analysis of recovered sequences. (**A**) Taxonomic assignment of CPN60 sequences from metagenomic-assembled contigs across control, water, and oyster samples, using both hybridization capture and shotgun metagenomics. Yellow indicates detection of CPN60 sequences assigned to each taxon; blue indicates not detected. Taxa include *Vibrio* species, primarily detected by hybridization capture sequencing, as well as non-*Vibrio* microbial taxa detected by shotgun metagenomics. (**B**) Neighbor-joining phylogenetic tree of *Vibrio* spp. CPN60 nucleotide sequences recovered from metagenomics (blue text, starred) and reference sequences (black text) from the chaperonin database. Red dots represent bootstrap support at key nodes. Tree illustrates the phylogenetic placement of environmental sequences among known *Vibrio* species, confirming species-level assignments and revealing diversity among recovered CPN60 sequences.

Gene profiling of the CPN60 marker was used to assess the diversity of *Vibrio* spp. within the metagenomic samples (Fig. 3). The taxonomic assignment of CPN60 sequences revealed the presence of multiple taxa, with water samples showing a greater diversity compared to oyster samples (Fig. 3A). Although shotgun metagenomics detected a higher overall number of taxa, *Vibrio* spp. were exclusively detected using HCS. Notably, *V. aestuarianus*, *V. vulnificus*, and *V. cholerae* were identified using HCS, whereas *Aphanothece* spp. and *Cyanobium* spp. were predominantly detected in shotgun metagenomic samples.

Phylogenetic analysis of the recovered CPN60 sequences (Fig. 3B) confirmed the taxonomic assignments of *Vibrio* spp., with the recovered sequences clustering within the expected clades, indicating consistency between environmental isolates and known *Vibrio* lineages. For all *Vibrio*-specific CPN60 assignments, except for *V. vulnificus* recovered from a water sample collected at BRO, the identified species were also detected using k-mer-based whole-genome profiling (Fig. 2D). These results highlight the utility of CPN60 as a robust taxonomic marker for profiling bacterial diversity in complex environmental samples.

#### Capturing antimicrobial resistance and virulence determinants

**Fig 4:**
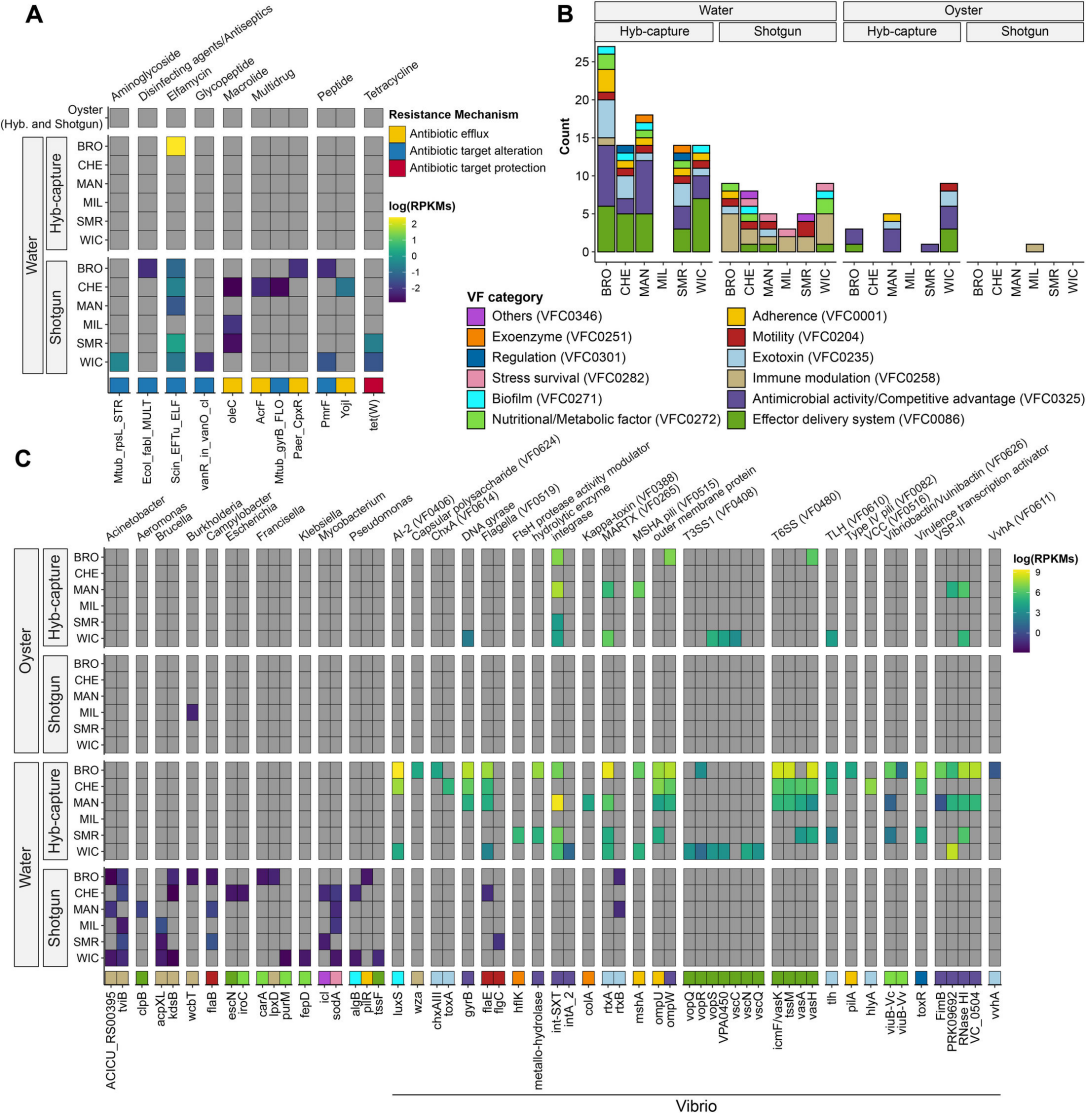
Detection and quantification of antimicrobial resistance and virulence factor-associated genes using ShortBRED. (**A**) Heatmap showing detection and quantification of antimicrobial resistance genes from the CARD, stratified by resistance mechanism. Color scale indicates log-transformed reads per kilobase per million mapped reads, log(RPKMs). (**B**) Bar plot showing the total count of virulence factor genes detected in each sample, grouped by virulence category based on annotations from the VFDB and probe targets included in the HCS panel. (**C**) Heatmap showing detection and quantification of virulence factors, including genes from VFDB and custom probe targets, across different sample types and sequencing methods. Virulence genes are grouped by genus (top) and functional category (bottom). Color scale represents log-transformed RPKMs, and VF categories are color-coded in the legend to indicate functional roles.

Antimicrobial resistance genes and virulence factors were identified from metagenomic samples using ShortBRED (88) and blastx (89). The detected antimicrobial resistance genes (ARGs), categorized by resistance mechanism, are shown in Fig. 4A. Genes related to antibiotic efflux (e.g., *oleC*, *AcrF*, *CpxR*, and *Yojl*), target alteration (e.g., *rpsL*, *fabL*, *EF-Tu*, vanR, gyrB, and PmrF), and target protection (tetW) were predominantly found in water samples processed via shotgun metagenomics, whereas HCS detected fewer ARGs overall. Notably, no ARGs were detected in oyster samples.

The distribution of detected VFs across sample types is shown in Fig. 4B and C. Overall, a greater number of VF genes were identified in water samples compared to oyster samples, with HCS consistently detecting more VFs than shotgun metagenomics. The detected VFs spanned several functional categories, including adherence, exoenzymes, biofilm formation, and competitive advantage, with genes characteristic of *Vibrio* spp. being particularly prevalent. Among samples profiled using HCS, several biomarkers for species detection and identification were identified, including *toxR* (genus *Vibrio*), *tlh* (*V. parahaemolyticus*), *vvhA* (*V. vulnificus*), and *ompW* (*V. cholerae*).

However, important determinants associated with clinical *V. cholerae* (*ctxA* and *rfb*-O1) and *V. parahaemolyticus* (*tdh* and *trh*) were not detected, despite the presence of the latter in WGS isolates from the sampling areas. Other VFs, such as those related to type III secretion system (T3SS) (type 1) and type VI secretion system (T6SS), exotoxins (rtxA, *chxAIII*, *toxA*, *rtxA*, *rtxB*, and *hlyA*), and exoenzymes (*hflk* and *colA*), were commonly identified. Additionally, genes associated with *V. cholerae* conjugative elements, namely, *Vibrio* seventh pandemic island II (VSP-II), were detected in most water samples.

It is also worth noting that the detection of non-*Vibrio* VFs was more frequently detected in samples analyzed via shotgun metagenomics, which aligns with previous observations from this study. This indicates that while HCS is highly efficient for detecting targets of interest, shotgun metagenomics captures a broader range of background microbial diversity, including ARGs and VFs.

#### Comparative genomics of *Vibrio* spp. isolates employing WGS

**Fig 5:**
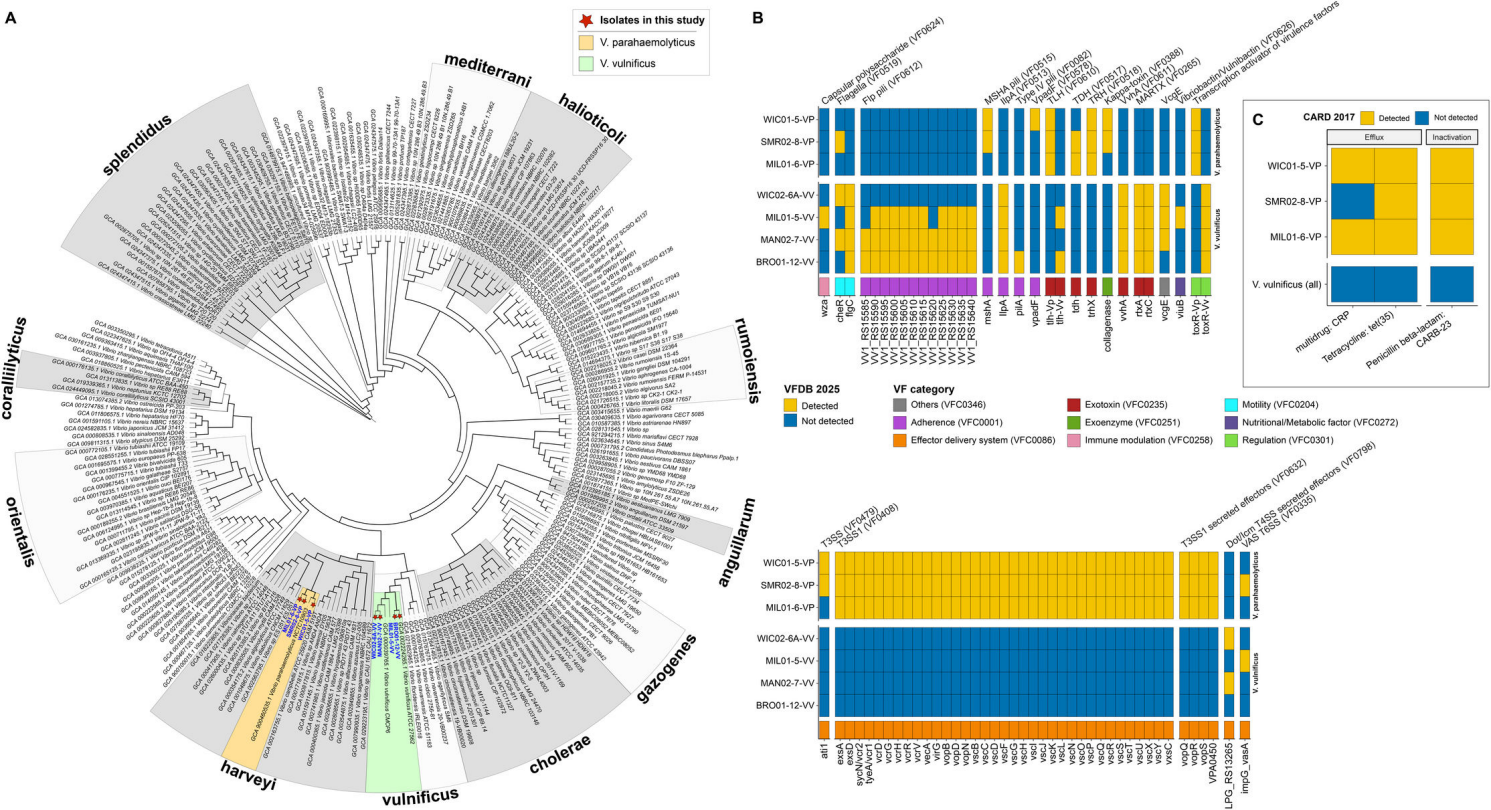
Phylogenomic relationships of *Vibrionaceae* isolates and characterization of antimicrobial resistance and virulence factor-associated genes using ShortBRED. (**A**) Maximum-likelihood phylogenetic tree of *Vibrionaceae* constructed using single-copy core genes identified by GToTree. Isolates recovered in this study are marked with red stars, and taxonomic groups of interest (*V. parahaemolyticus* and *V. vulnificus*) are highlighted in orange and green, respectively. (**B**) Heatmaps showing detection of VFs in *Vibrio* spp. isolates. VFs are grouped by functional category according to the VFDB and probe targets included in the HCS panel, with color-coding for functional role. (**C**) Detection of antimicrobial resistance genes from the CARD database, grouped by resistance mechanism. Yellow indicates the presence, and blue indicates the absence of the target gene or protein family.

The seven *Vibrio* spp. isolates were subjected to WGS, and comparative genomics with 233 representative *Vibrio* spp. genomes present in the GTDB (94) was performed. A search for homologous genes returned 355 SCGs (ca. 8.05 × 10^4^ amino acids in length) shared among the genus *Vibrio*. Within the *Vibrionaceae* phylogeny, each species formed coherent clusters in taxonomic subclades (Fig. 5A). Four isolates obtained in this study formed a distinct cluster with representative *V. vulnificus* strains. Three isolates clustered with representative *V. parahaemolyticus*, the latter forming a subclade within the *V. harveyi* group that also included *V. alginolyticus*, *V. diabolicus*, *V. campbellii*, *V. owensii*, and *V. harveyi*.

**Fig 6:**
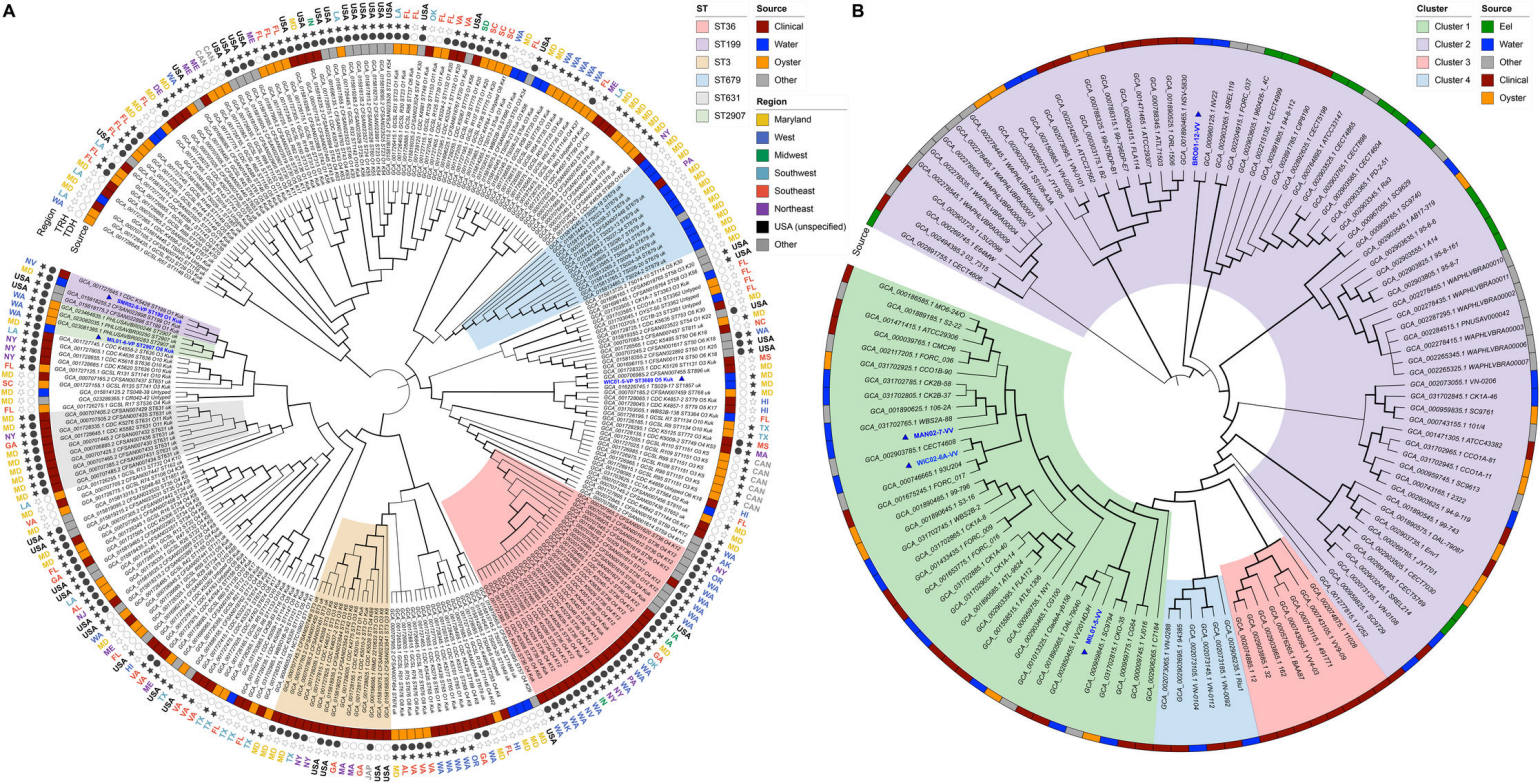
Phylogenetic relationships of *Vibrio* species isolates recovered in this study. Maximum-likelihood phylogenetic tree of (**A**) *Vibrio parahaemolyticus* (*n* = 259 genomes) and (**B**) *Vibrio vulnificus* (*n* = 118 genomes), constructed using single-copy core genes identified by GToTree. Isolates recovered in this study are highlighted in blue and denoted with triangles. Trees were rooted and visualized using iTOL. Clustering illustrates the genomic relatedness of environmental isolates from this study relative to globally distributed reference strains, supporting lineage classification and potential virulence associations.

Using a homologous search of the *Vibrio* SCG set, phylogenomic trees were also built for *V. parahaemolyticus* (Fig. 6A) and *V. vulnificus* (Fig. 6B). Using alleles for *dnaE*, *gyrB*, *recA*, *dtdS*, *pntA*, *pyrC*, and *tnaA*, the three *V. parahaemolyticus* isolates were each assigned to different MLST profiles (ST199, ST2907, and ST3669). None of the isolates were assigned to clonal complexes (Table S11). Based on the phylogeny of the *Vibrio* SCG set for *V. parahaemolyticus*, well-defined clades for sequence types primarily of clinical origin were observed, including ST3, ST36, and ST631. While *V. parahaemolyticus* isolates from this study encoded *tdh*/*trh*, they clustered most closely with other environmental isolates. WIC01-5 clustered closely with an isolate recovered previously from Tangier Sound, Maryland, while MIL01-6 clustered with oyster isolates from Washington and SMR02-8 with a clinical isolate from Nevada. Of the four *V. vulnificus* isolates, WIC02-6A was assigned to ST286, and the remaining isolates were not assigned to an established MLST profile, using alleles for *glp*, *gyrB*, *mdh*, *metG*, *purM*, *dtdS*, *lysA*, *pntA*, *pyrC*, and *tnaA* (Table S12). Following *Vibrio* SCG phylogeny, *V. vulnificus* strains clustered into four distinct groups (C1 to C4). C1 and C2 comprised most of the genomic diversity, while isolates within C3 and C4 were more closely related to each other. Three isolates were phylogenetically joined in C1 and one in C2. While both clinical and environmental isolates were present in distally related clades, isolates from this study paired most closely with those of environmental origin.

#### Genetic characterization of *Vibrio* isolates

Profiling *Vibrio* spp. isolates using ShortBRED (88) and blastx (89) yielded important information concerning the presence of VFs (Fig. 5B) and ARGs (Fig. 5C). Species identity was supported by the presence of species-specific virulence regulator *toxR* in all isolates. In addition, VF genes associated with adherence, exotoxin production, and exoenzyme activity were commonly detected across isolates.

Among the *V. parahaemolyticus* isolates, the virulence markers *tlh* and the T3SS (type 1) were consistently present. Consistent with PCR results (Fig. 1B), two *V. parahaemolyticus* isolates carried the thermostable direct hemolysin (*tdh*) gene, and all encoded the *tdh*-related (*trh*) gene. In addition, one *V. parahaemolyticus* isolate carried *impG/vasA,* suggesting the presence of the T6SS.

All *V. vulnificus* isolates encoded the hemolysin gene *vvhA* and RTX toxin. In addition, genes associated with iron acquisition, such as *vcgE*, vulnibactin (*viuB*), and *pilA*, were detected, along with the *V. vulnificus* homolog of *tlh*. Two *V. vulnificus* isolates also encoded the type IV secretion system, while one isolate carried T6SS.

Regarding antimicrobial resistance, all *V. parahaemolyticus* isolates carried ARGs (Fig. 5C), notably genes conferring resistance to tetracycline (*tet35*) and penicillin (*CARB-23*). Additionally, two *V. parahaemolyticus* isolates harbored the multidrug resistance gene *CRP*, indicating potential resistance to multiple antibiotic classes. In contrast, no ARGs were detected in the *V. vulnificus* isolates.

These findings indicate that *V. parahaemolyticus* isolates in this study possess a greater potential for antimicrobial resistance compared to *V. vulnificus* isolates. However, all isolates in this study harbored a broad range of VFs, highlighting the potential for diverse pathogenic mechanisms within environmental *Vibrio* populations in the Chesapeake Bay.

## DISCUSSION

*Vibrio* spp. are an integral part of the microbial flora in aquatic environments globally, particularly within crustaceans and zooplankton. Environmental factors influence the proliferation of *Vibrio* spp. and their transmission through food and water. Climate parameters, in particular, have been linked to the increased proliferation of pathogenic *Vibrio* spp., correlating with a rise in the number of infections caused by these bacteria globally (3, 4, 8, 15, 109–114).

Given the ecological significance of *Vibrio* spp., including their commensal and mutualistic interactions with multicellular hosts, controlling human infections caused by these bacteria requires accurate surveillance and effective public health interventions. Early warning systems that identify conditions conducive to growth and proliferation of pathogenic strains in the environment are essential for mitigating infection risks. Development of such systems will require a multidisciplinary integration approach, integrating knowledge of environmental factors that promote the persistence of pathogenic agents with sociological and behavioral factors facilitating human interactions with those pathogens.

Surveillance of the incidence and virulence profiles of pathogens in the environment is crucial for informing and training predictive models. Historically, detecting vibrios in the environment has been challenging due to their ability to enter a VBNC state, which complicates detection using traditional culture-based methods and can lead to underestimation of risk. Advanced molecular techniques offer a more detailed understanding of *Vibrio* spp. population dynamics, providing valuable data that can be incorporated into predictive models to enhance early warning systems.

Investigation of pathogenic *Vibrio* spp. has been ongoing in the Chesapeake Bay since the 1960s (6–8). Studies have shown that *Vibrio* spp. thrive in warm water with moderate salinity. However, detecting and typing *Vibrio* spp. have been done primarily for *V. cholerae*, *V. parahaemolyticus*, and more recently *V. vulnificus*. Recent assessment of the microbial community composition of blue crabs and water from Maryland coastal regions confirmed frequent detection of both *V. parahaemolyticus* and *V. vulnificus* by employing real-time PCR (115). However, detection of *Vibrio* spp. was infrequent when employing 16s rRNA amplicon sequencing. Furthermore, other important biomarkers could not be identified. Shotgun metagenomic sequencing provided a method for studying the total genetic material of a sample and proved useful in identifying pathogenic *Vibrio* spp., especially in clinical samples, including cholera (36) and vibriosis patients (37). However, when shotgun metagenomics is applied to environmental samples, the number of reads profiled as *Vibrio* spp. is often not sufficient for highly specialized applications, such as comparative analyses or threat assessment and attribution (15). A primary reason is that pathogenic agents frequently may be present only at low levels relative to the total microbial population of a given sample, particularly in shotgun metagenomic analysis of oyster samples, since sequencing read libraries are dominated by the host DNA, thereby complicating metagenomic profiling (116).

In this study, an alternative method for *Vibrio* spp. detection and characterization has been devised by creating targeted HCS assays of 69 *Vibrio* spp. and 162 virulence markers (Table S1). To confirm its value, the method was employed, along with traditional culture WGS and whole-community shotgun metagenomic sequencing, to profile the microbiome of water and oyster samples collected from the Chesapeake Bay.

Employing k-mer alignment to whole-genome reference databases (Fig. 2), HCS captured a greater diversity of *Vibrio* spp. compared to shotgun metagenomics. The probes designed for the enrichment panel, which included both CPN60 and *Vibrio*-specific biomarkers, enhanced homologous genomic regions of *Vibrio* spp. as well as the intended targets. One unexpected finding was the lack of significant differences in alpha diversity between the two sequencing methods (Fig. 2A). It was anticipated that preferential enrichment of *Vibrio* spp. targets would reduce overall diversity in HCS samples. However, this outcome may be due to the inherent nature of HCS, which does not fully deplete non-target sequences but instead increases the abundance of specific targets while still capturing background diversity from abundant taxa. Additionally, HCS increased the number of sequencing reads suitable for taxonomic profiling in oyster samples, which were predominantly composed of *Synechococcus* and *Vibrio*, consistent with previous studies (117). In contrast*,* shotgun sequencing detected a greater number of non-target taxa, notably viruses. This indicates that while HCS improved the sensitivity of *Vibrio* detection, it does not completely exclude non-target organisms, resulting in diversity estimates comparable to those obtained through shotgun sequencing.

Metagenomic analysis of a mock community (Fig. S4) revealed that shotgun sequencing most accurately reflected the expected relative abundance profiles, while HCS showed a bias toward *V. cholerae*. This bias was anticipated, as many of the probes were specifically designed to target well-characterized virulence factors in *V. cholerae*, particularly the Affertcholeramvirus CTXφ. Although HCS increased the detection sensitivity of *Vibrio* spp., this inherent bias indicates that the method is not currently suitable for accurate quantification due to targeted enrichment.

Among the 10 representative *Vibrio* spp. included in the mock community, 9 were correctly identified using shotgun metagenomics, and 8 were detected using HCS, based on k-mer alignment and whole-genome databases. These observations were further supported by the taxonomic assignment of CPN60 sequences recovered from metagenomic contigs, which proved to be more accurate at profiling the mock community. Notably, *V. alginolyticus* was identified as *V. diabolicus*/*Vibrio* sp. Ex25, which are taxonomic synonyms for *V. alginolyticus* (118). In addition, *V. fischeri* was not detected using either method, likely due to the absence of a high-quality genome sequence for *Aliivibrio* (*Vibrio*) *fischeri* ATCC 25918 in public repositories, resulting in its incomplete representation in the reference databases.

Due to the study design, the limit of detection could be estimated only at approximately 0.6% relative sequencing read abundance for species detection. However, previous research reported HCS targeting the 16S rRNA gene achieved a much lower limit of detection, around 0.00006% relative abundance (119). Further validation studies are needed to assess the sensitivity of the HCS approach developed here under different environmental contexts, since the lack of detection of certain genes may result from either natural environmental variability or methodological limitations.

Use of the CPN60 marker in metagenomic analysis provided valuable insight into the diversity of *Vibrio* spp. within environmental samples, highlighting the effectiveness of HCS in detecting specific taxa. Findings of CPN60 profiling of metagenomic contigs revealed that, while shotgun metagenomics identified a higher overall number of taxa, *Vibrio* spp. were exclusively detected using HCS (Fig. 3). Among the *Vibrio* spp. identified, *V. aestuarianus*, *V. vulnificus*, and *V. cholerae* were detected using HCS, while other non-*Vibrio* taxa such as *Aphanothece* spp. and *Cyanobium* spp. were predominantly observed with shotgun sequencing. This discrepancy between methods can be attributed to the targeted nature of HCS, compared to shotgun metagenomics which provides a broader snapshot of microbial diversity. In addition, phylogenetic analysis of the recovered CPN60 sequences further validated taxonomic assignments (Fig. 3B). Notably, all *Vibrio*-specific CPN60 assignments, except for a *V. vulnificus* sequence, were also detected using k-mer-based whole-genome profiling (Fig. 2D). These findings demonstrate the utility of CPN60 as a reliable marker for *Vibrio* spp. profiling, particularly when combined with HCS for enhanced detection sensitivity.

Comprehensive metagenomic analysis revealed ARGs and VFs were more commonly detected in water samples compared to oyster samples, with HCS generally identifying more VFs while shotgun metagenomics detected more ARGs (Fig. 4). This difference can be attributed to the design of the HCS probes, which specifically targeted *Vibrio* VFs. Other targeted capture protocols developed for ARGs, e.g., (46), will be considered in our future studies to improve antimicrobial resistance profiling in complex samples.

The identified VFs encompassed a range of functions, including adherence, exoenzymes, biofilm formation, and competitive advantage, with *Vibrio*-specific markers such as *toxR*, *tlh*, *vvhA*, and *ompW* being prevalent in samples analyzed using HCS. However, the primary VFs of *V. parahaemolyticus* (*tdh* and *trh*) were not detected using HCS, despite being present in WGS isolates from the sampling areas. Similarly, *tlh* and *vvhA* were detected more commonly by PCR (Fig. 1B) than by metagenomic methods. Consistent with previous findings, non-*Vibrio* VFs were more frequently detected using shotgun metagenomics compared to HCS.

*V. parahaemolyticus* and *V. vulnificus* isolates were recovered from water samples collected during a comprehensive study done during 2019 in the Chesapeake Bay (4) and subjected to WGS for phylogenomic analysis (Fig. 5 and 6). Phylogenetic clustering revealed multiple clonal populations circulating simultaneously, rather than all isolates from the same region clustering together. This pattern aligns with previous analyses of *Vibrionaceae* (120), *V. parahaemolyticus* (121–123), and *V. vulnificus* (124, 125). Among the *V. parahaemolyticus* isolates, two carried both *tdh* and *trh*, while one carried only *trh*. Notably, the pandemic complex (ST3, O3:K6) and prevalent clinical strains in North America, e.g., ST36 (O4:K12) and ST631, were not detected. However, one isolate profiled as ST199 had been previously reported from clinical cases in the USA (121) and China (126). *V. vulnificus* isolates classified into clusters C1 and C2, which have been reported elsewhere (124). All *V. vulnificus* isolates encoded the environmental biotype of the virulence-correlated gene (*vcgE*), although recent evidence suggests that biotype alone is not a reliable predictor of virulence potential (127, 128). Compared to *V. parahaemolyticus*, fewer *V. vulnificus* isolates from the Chesapeake Bay have been sequenced, with most available genomes originating from clinical sources. The genetic diversity observed in this study suggests that the Chesapeake Bay may serve as a reservoir of *Vibrio* genetic variability*,* highlighting the need for further genomic investigations.

While traditional culture-based methods proved useful for recovery of pathogenic *V. parahaemolyticus* and *V. vulnificus* from the Chesapeake Bay, it should be noted that the majority of *V. parahaemolyticus* isolates recovered from the Chesapeake Bay did not encode *tdh* and/or *trh* (4, 129). Hence, while pathogenic *Vibrio* spp. are clearly present in the Chesapeake Bay, culture isolation-based studies do not reflect the complex dynamics of microbial communities accurately. In contrast, culture-independent molecular methods, namely shotgun metagenomics and HCS, effectively profile the microbiome, allowing more accurate detection and characterization of *Vibrio* spp.

### Challenges and future directions

Currently, PCR is routinely employed for biomarker detection and has proven both practical and useful for infectious disease diagnostics and for some aspects of environmental surveillance by circumventing issues associated with culturing. In this study, PCR detected *V. cholerae*, *V. parahaemolyticus*, and *V. vulnificus* (Fig. 1B). However, PCR is limited in its ability to genetically profile samples since it only targets one or a few markers at a time. Except for a single water sample, whole-community shotgun metagenomic sequencing, performed at the depth used in this study, was insufficient to profile sequencing reads to members of the genus (Fig. 2D). In contrast, the HCS method demonstrated the presence of *Vibrio* spp. and revealed that carriage of *Vibrio*-specific VFs was more common than previously recognized. As expected, shotgun metagenomics of both water and oyster samples identified a wider range of species and other taxa, notably viruses, that were not detected by HCS.

The most time-consuming aspect of this study was the initial design and optimization of the HCS panel. Now that the panel has been developed, incorporating it into traditional NGS workflows adds approximately 1 additional day. Despite this added time, the method offers significant advantages by enabling detection of a broader range of target species and genes without the need to screen each target individually. The probe targets were specifically designed to keep the total number of probes low (less than 2,000, i.e., the minimum panel design available for most commercial suppliers), thereby reducing costs and making the method more accessible while still achieving a broad depth of targets.

Clearly, HCS can be further enhanced in probe design. Although HCS presently takes longer to perform than other sequencing methods (Fig. S1), it achieves much greater analytical sensitivity using fewer sequencing reads, significantly reducing sequencing cost when the targets of interest are known (130). With panels developed for *Vibrio* pathobiota of oysters (44), as well as 16S rRNA (45) and antimicrobial resistance genes (46), hybridization capture bait sets can be scaled rapidly. Additionally, selective culture enrichment using APW facilitates the identification of diverse elements and amplification of *Vibrio* spp. (15, 22, 131). Hence, HCS coupled with enrichment offers a promising area for further improvement.

One unexpected finding was the absence of *tdh* and *trh*, important *V. parahaemolyticus* VFs, in HCS data, despite their presence in WGS samples (Fig. 1B). This discrepancy suggests that while HCS improves detection sensitivity for specific targets, it may still miss certain critical markers. Thus, PCR, shotgun metagenomic sequencing, and HCS should be considered complementary rather than interchangeable methods of comprehensive environmental surveillance.

Although this study reports results for a small number of samples without duplication, the results clearly reveal the presence of pathogenic *Vibrio* spp. *V. cholerae*, *V. parahaemolyticus*, *V. vulnificus*, *V. fluvialis*, and *V. aestuarianus*, all of which are known causative agents of vibriosis, notably reported cases in Maryland (14). Additional studies in progress will characterize microbial communities in addition to *Vibrio* to provide a baseline of pathogenic agents present in water and oyster samples. Seasons of the year need to be studied as well to shed light on shifts in *Vibrio* populations annually and illuminate the spectra of microbiomes relative to changing environmental conditions. Predictive models will benefit from the inclusion of data from WGS, shotgun metagenomics, and HCS, particularly for pathogen surveillance, evolutionary analysis, and tracking the emergence of VFs and antibiotic resistance, as well as source attribution and outbreak monitoring.

## Data Availability

Sequencing data generated for all samples included in this study have been deposited in the NCBI Sequence Read Archive database under BioProject ID PRJNA1188529. Probe sequences for the custom *Vibrio* spp. hybridization panel (Tables S1 through S3), cpn60 sequences recovered from metagenomic assembled contigs (Table S7), and accession numbers for metagenomic sequencing libraries (Table S9) and draft genome assemblies (Table S10) are provided in the supplemental material.

## SUPPLEMENTAL MATERIAL

Supplemental figures and tables mbio.00516-25-s0001.xlsx Figures S1 to S5 and Tables S1 to S12.

ASM does not own the copyrights to Supplemental Material that may be linked to, or accessed through, an article. The authors have granted ASM a non-exclusive, world-wide license to publish the Supplemental Material files. Please contact the corresponding author directly for reuse.
